# Sweat, tears, and beyond: advanced wearable sensors for personalized health and athletic performance

**DOI:** 10.3389/fbioe.2025.1684674

**Published:** 2025-11-28

**Authors:** Erkan Gulgosteren, Sermin Agrali Ermis, Aydolu Algin Toros, Turhan Toros, Emre Serin, Mustafa Onder Sekeroglu, Numan Bahadır Kayisoglu

**Affiliations:** 1 Faculty of Sports Science, Mersin University, Mersin, Türkiye; 2 Faculty of Sport Sciences, Aydin Adnan Menderes Universitesi, Aydın, Türkiye; 3 Akdeniz Universitesi, Antalya, Türkiye; 4 Faculty of Sport Sciences Department of Coaching Education, Mersin University, Mersin, Türkiye; 5 Faculty of Sport Sciences, Mus Alparslan University, Muş, Türkiye; 6 Hasan Dogan Faculty of Sport Sciences, Karabuk University, Karabük, Türkiye

**Keywords:** wearable sensors, body fluid monitoring, non-invasive technologies, advanced materials, sports and healthcare applications

## Abstract

This review investigates the transformative potential of wearable sensors for body fluid monitoring in sports and healthcare. These devices offer a non-invasive, real-time, in-situ glimpse into our health by continuously tracking vital biomarkers found in sweat, saliva, urine, and tears. We discuss various sensor technologies, including electrochemical, optical, and microfluidic, and the innovative materials like hydrogels and nanocomposites that enable their functionality. The integration of RFID and flexible electronics is also examined, highlighting how these advancements improve the connectivity, portability, and user-friendliness of the sensors. Moreover, we delve into the advanced manufacturing techniques, such as 3D printing, that are crucial for crafting these sophisticated devices with high precision and adaptability. In prospect, this article evaluates the transformative potential of integrating these in-situ sensors with artificial intelligence and machine learning, envisioning a paradigm shift in health monitoring and athletic performance optimization.

## 1 Introduction

The integration of wearable sensor technologies into sports and health monitoring marks a significant evolution in personalized healthcare and athletic performance. These devices transcend the limitations of traditional diagnostic methodologies, which are inherently constrained by their reliance on invasive, episodic, and laboratory-based analyses. By leveraging advancements in flexible materials, sophisticated electronics, and wireless communication protocols, wearable sensors facilitate the continuous and unobtrusive monitoring of physiological states. This capability not only democratizes access to health data but also empowers individuals with real-time insights, fostering proactive health management. Such advancements are particularly salient in sports science, where nuanced physiological data underpin performance optimization and recovery strategies, and in healthcare, where early intervention and the longitudinal management of chronic conditions are paramount.

The market dynamics surrounding wearable health sensors and sports devices reflect a robust growth trajectory. Projections indicate a 10.5% Compound Annual Growth Rate (CAGR) for the Wearable Health Sensors Market, driven primarily by remote patient monitoring applications that capitalize on continuous health data acquisition and analysis. Similarly, the wearable sports device market is poised for expansion, from an estimated USD 94.17 billion in 2025 to USD 115.57 billion by 2030, representing a 4.18% CAGR. This surge is propelled by the sports industry’s increasing reliance on real-time athletic performance and health metrics (Mordor Intelligence, 2025a; Mordor Intelligence, 2025b). The confluence of technological innovation, enhanced device comfort and portability, and heightened health and fitness awareness underpins this growth. These trends not only accelerate the adoption of wearable technologies but also facilitate their seamless integration into daily life, thereby redefining preventive healthcare and sports training paradigms. The increasing embedment of these devices within consumer and medical ecosystems heralds a shift towards data-driven personal and professional health decision-making.

Within the realm of sports and health monitoring, a diverse array of sensors—including Electrocardiography (ECG), Electromyography (EMG), Functional Near-Infrared Spectroscopy (fNIRS), and Inertial Measurement Units (IMU)—contributes to the optimization of athletic performance and the safeguarding of wellbeing (Seçkin et al., 2023). Notably, sensors designed for direct interaction with body fluids occupy a pivotal role. For instance, Pulse Oximeter sensors measure blood oxygen saturation (Seçkin et al., 2023), providing critical insights into respiratory efficiency and cardiovascular function. Blood Pressure Sensors (BPS) enable non-invasive monitoring of blood pressure, offering essential data on cardiovascular health, particularly during strenuous physical activity (Iqbal et al., 2024). Galvanic Skin Response (GSR) sensors (Mao et al., 2023) detect variations in skin conductivity due to perspiration, revealing stress levels and physiological responses to training.

Wearable body fluid sensors, specifically, distinguish themselves through their capacity to provide real-time, direct biomarkers of an athlete’s physiological state, enabling tailored training regimens and health monitoring. Building upon this foundation, wearable sensor technology has revolutionized health monitoring and athletic performance assessment through non-invasive, real-time tracking of critical physiological parameters. The capacity to analyze extracellular body fluids—including blood, sweat, saliva, urine, and tears—has yielded unprecedented insights into biomarkers such as pH, glucose, electrolytes, osmolality, and cortisol. These advancements have redefined approaches to metabolic health management, hydration strategies, stress response monitoring, and athletic optimization, paving the way for personalized healthcare and performance-driven strategies (Kristoffersson and Lindén, 2022; Kelly et al., 2024; Magdalena, 2024; Obaidur Rahman Khan et al., 2024; Jafleh et al., 2024; Vishnupriya, 2024; Wenfei, 2024).

Each body fluid presents unique characteristics and analytical challenges (Ates et al., 2021), dictating its suitability for wearable sensor applications. While blood offers a comprehensive biomarker profile and remains the gold standard for many clinical diagnostics, its invasive sampling requirements limit its practicality for continuous monitoring (Islam and Washington, 2024). Non-invasive alternatives—sweat, saliva, urine, and tears—offer significant advantages in accessibility and ease of use (Kim et al., 2019). Sweat provides biomarkers such as sodium, lactate, and cortisol, offering crucial insights into hydration and metabolic states (Min et al., 2023; Childs et al., 2024; Baker, 2017). Saliva serves as a convenient matrix for monitoring stress, immune function, and glucose levels (Chojnowska et al., 2021; Pfaffe et al., 2011; Kumari et al., 2024). Urine provides a cumulative reflection of hydration, renal health, and electrolyte balance (Xue et al., 2023; Wardenaar, 2022). Tears, with biomarkers such as osmolality and cortisol, offer a unique avenue for assessing ocular and systemic health (Potvin et al., 2015; Alkozi et al., 2024).

This transition signifies a transformative era in personalized healthcare and sports science, wherein in-situ wearable sensors facilitate precise real-time health monitoring, enhanced athletic performance insights, and early intervention capabilities. By enabling continuous data collection directly from the body, these sensors obviate the need for laboratory-based testing, offering unparalleled convenience and accuracy. The ensuing discussion will delve into the specific properties and applications of body fluids in wearable sensor technologies, addressing the challenges and innovations that are shaping the future of this dynamic field. Although many reviews have addressed individual biofluid sensors, few have systematically compared cross-fluid integration (blood–sweat–tear) within AI/IoT frameworks for athletic optimization and personalized healthcare.

## 2 Body fluids key sensor parameters

Wearable sensors for body fluid monitoring represent the intersection of technological innovation, scientific discovery, and practical utility. Their ability to provide continuous, non-invasive access to critical health metrics empowers individuals and professionals to make informed decisions, transforming the landscape of healthcare and performance optimization. The integration of these sensors into body fluid analysis has enabled precise and dynamic monitoring of key physiological parameters vital to health and performance. Among these, pH (Heikenfeld et al., 2018; Wolf et al., 2011), conductivity (Shirreffs and Maughan, 1998), dielectric constant (Wolf et al., 2011), and biomarker-specific metrics (Gao et al., 2021; Koh et al., 2016; Jaiswal et al., 2024) stand out as particularly informative. These parameters not only reflect the body’s immediate physiological state but also provide insights into long-term health trends. Advances in sensor technologies have enabled the development of highly sensitive and non-invasive devices capable of detecting subtle changes in these metrics, offering actionable insights for both healthcare practitioners and athletes. The ability to monitor these parameters in real time allows users to make timely adjustments to hydration, nutrition, and recovery strategies, minimizing risks and maximizing performance. Exploring the significance and applications of these parameters underscores how wearable sensors are reshaping the understanding and utilization of body fluid analytics in healthcare and sports science.

Among the various parameters, pH stands out as a critical measure of the acid-base balance in body fluids, essential for maintaining physiological stability and overall health. For athletes, pH monitoring in sweat, blood, urine, or tears reveals valuable data about metabolic processes, hydration levels, and recovery efficiency. Wearable sensors for pH measurement, employing technologies like electrochemical systems or optical methods, provide real-time tracking of acid-base shifts. Such insights allow timely interventions to mitigate fatigue, dehydration, or metabolic stress, enhancing performance and recovery. Non-invasive pH sensors, designed to be flexible and biocompatible, monitor various physiological parameters by detecting ionic changes in sweat (Hossain et al., 2023), which indicate the body’s metabolic state. When integrated with IoT platforms such as Blynk App (Sathya and Rajalakshmi, 2023) and AI analytics, these sensors promise to enhance personalized training and hydration strategies while addressing potential health risks related to pH imbalances.

Similarly, monitoring the conductivity of body fluids, linked to ion concentration, offers significant insights into hydration and electrolyte balance. Conductivity sensors integrated into wearable devices detect ionic changes, providing real-time data crucial for managing hydration and preventing imbalances. These compact, non-invasive sensors have gained traction in sports and health applications, and their future integration with IoT and AI is poised to enhance personalized electrolyte management, ultimately improving athletic performance and health outcomes.

The dielectric constant, indicative of a fluid’s ability to store electrical energy, further enhances the understanding of body fluid composition. By monitoring the dielectric constant of sweat, blood, saliva, or tears, wearable sensors offer critical insights into hydration, electrolyte balance, and overall physiological health. Advances in sensor technology, such as RF and capacitive systems, enable highly sensitive, real-time, and non-invasive monitoring. These capabilities provide athletes with actionable data to optimize performance and recovery while reducing risks associated with dehydration and electrolyte imbalances.

Electrolyte monitoring is another essential application of wearable sensors. Electrolytes such as sodium, potassium, calcium, and chloride are critical for hydration, nerve function, and muscle performance. Wearable sensors, employing advanced electrochemical and ion-selective technologies, allow real-time tracking of electrolyte levels, offering athletes precise data to adjust hydration and nutrition strategies. The integration of these sensors with IoT and AI systems ensures a personalized approach to optimizing health and performance, reducing risks associated with electrolyte imbalances.

Osmolality, a measure of solute concentration, is crucial for assessing hydration and metabolic health (Shirreffs and Maughan, 1998). Innovative sensor technologies, including microfluidic, conductivity-based, and optical systems, provide precise osmolality measurements, often integrated into wearable devices for real-time tracking. These advancements facilitate early detection of dehydration or overhydration and help maintain fluid and electrolyte balance, ensuring optimal performance. As sensor technologies evolve, non-invasive approaches using sweat, saliva, and tears will continue to improve, supported by IoT integration and AI-driven analysis for comprehensive health management.

**FIGURE 1:**
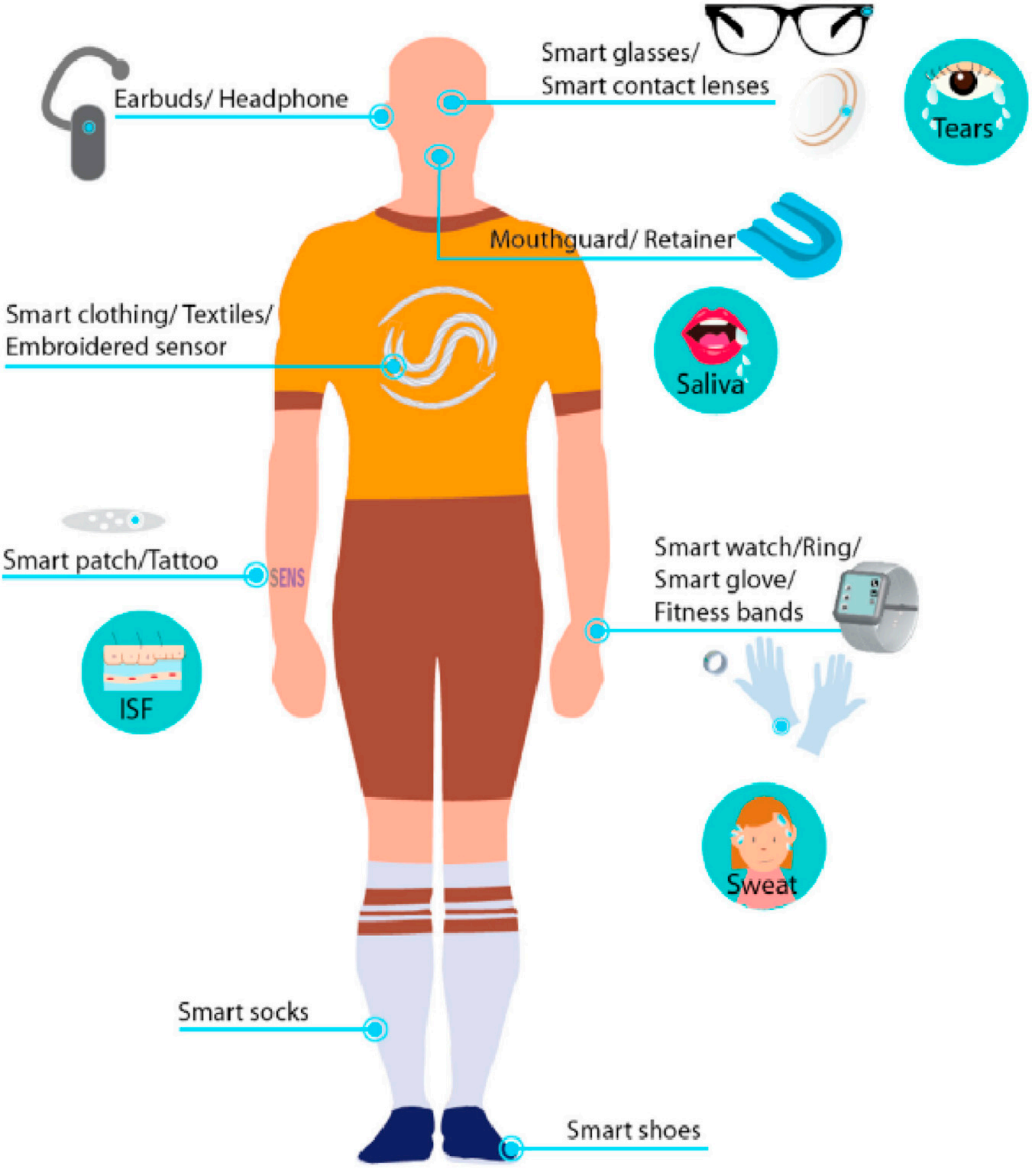
Depiction of various non-invasive chemical sensors applied to the human body, illustrating the diverse applications and sensor technologies used for monitoring different physiological parameters. (ISF: Interstitial fluid) (Promphet et al., 2021).

Recent advancements in materials science and manufacturing have played a pivotal role in enabling the integration of wearable sensors with body fluids (Fakhr et al., 2024; Promphet et al., 2021; Yuan et al., 2022; Wu et al., 2022; Shahzad et al., 2024). The use of flexible substrates like polydimethylsiloxane (PDMS) and advanced conductive materials such as graphene and silver nanowires has led to the development of lightweight, biocompatible sensors. Hydrogels, enhancing biofluid interaction, ensure reliable biomarker detection even under dynamic conditions (Zazoum et al., 2022). Furthermore, wireless communication technologies, including RFID and Bluetooth, have expanded the scalability and usability of these devices, allowing seamless data collection and analysis. Table 1 provides examples of body fluids, including tears, and their parameters, highlighting their impact on an athlete’s body and the potential sensor types that can be used for detecting health issues, as documented in the literature. Figure 1 also shows some non-invasive chemical sensors used on the human body (Promphet et al., 2021).

**TABLE 1:** Body Fluids, Measurement Parameters, In-situ Sensors, and Their Impact on Athletes’ Health: Wearable/nonwearable samples.

Body fluid	Parameter	Normal range	Issues with excess	Issues with deficiency	Potential issues in athletes	Sensor used	Sensor type	Attachment site(s)	References
Blood	pH	7.35–7.45	Alkalosis: Headache, muscle spasms	Acidosis: Respiratory failure, fatigue	Performance decline, muscle spasms	i-STAT System, Medtronic	Electrochemical Sensor	Fingerstick	Heikenfeld et al. (2018), Wolf et al. (2011)
	Conductivity	0.7–0.9 S/m	Hypernatremia: Increased blood pressure	Hyponatremia: Muscle cramps, endurance loss	Electrolyte imbalance, fatigue	Orion Conductivity Probe	Conductivity Sensor	Finger, intravenous	Shirreffs and Maughan (1998)
	Dielectric Constant	58–62	Increased cell membrane permeability	Slowed neural communication	Cellular communication disruption	Agilent E4991B Dielectric Test Fixture	Dielectric Spectrometer	Research laboratory	Wolf et al. (2011)
	Osmolality	∼290 mOsm/kg	Hyperosmolality: Dehydration	Hypoosmolality: Hyponatremia	Muscle fatigue, recovery challenges	Advanced Instruments Osmometer	Osmometer	Finger, intravenous	Shirreffs and Maughan (1998)
	Glucose	70–140 mg/dL	Hyperglycemia: Performance fluctuations	Hypoglycemia: Energy deficiency	Endurance decline, concentration issues	Dexcom G6, FreeStyle Libre	Continuous Glucose Monitor	Arm, abdomen	Gao et al. (2021)
	Lactate	<2 mmol/L (resting)	High lactate: Muscle fatigue	Low lactate: Insufficient energy production	Slowed muscle recovery	Lactate Plus Analyzer	Lactate Analyzer	Finger, earlobe	Koh et al. (2016)
	Electrolytes	-	Hypernatremia/hyperkalemia: Increased blood pressure	Hyponatremia/hypokalemia: Muscle cramps	Electrolyte imbalance	Abbott i-STAT Electrolyte Panel	Electrochemical Sensor	Finger, intravenous	Alizadeh et al. (2018)
	Proteins	6–8 g/dL	Inflammation, liver damage	Reduced immune function	Prolonged recovery	Bio-Rad Protein Assay Kit	Optical Spectrometer	Research laboratory	Gao et al. (2021)
	Hormones	-	High cortisol: Stress	Low testosterone: Energy deficiency	Difficulty in stress management	Siemens Immulite Hormone Analyzer	Immunoassay Sensor	Research laboratory	Karachaliou et al. (2023)
	pH	4.0–6.8	Increased sweat acidity: Skin irritation	Electrolyte loss	Impaired thermoregulation	Gatorade GX Patch	Microfluidic Sensor Patch	Arm, back	Koh et al. (2016)
Sweat	Conductivity	0.2–0.6 S/m	High salt loss	Electrolyte deficiency	Muscle spasms	Eccrine Sweat Conductivity Sensor	Conductivity Sensor	Arm, back	Bron et al. (2007)
	Electrolytes	-	Excessive salt loss: Muscle cramps	Sodium/potassium deficiency	Thermoregulation issues	Wearable Electrolyte Patch	Electrochemical Sensor	Arm, back	Alizadeh et al. (2018)
	pH	6.2–7.6	Dry mouth, increased cavity risk	Oral infections, bad breath	Hydration affecting endurance	SalivaCheck Buffer	Chemical Test Strip	Oral cavity	Alizadeh et al. (2018)
Saliva	Cortisol	<15 ng/mL	High cortisol: Stress and fatigue	Low cortisol: Recovery insufficiency	Excessive stress, inadequate recovery	Salimetrics Cortisol ELISA Kit	Immunoassay Sensor	Oral cavity	Iqbal et al. (2023)
	pH	4.5–8.0	Urinary tract infection	Kidney stone risk	Muscle cramps, dehydration	pH Indicator Strips	Chemical Test Strip	Urine sample	Alizadeh et al. (2018)
Urine	Conductivity	0.1–0.2 S/m	Electrolyte imbalance	Insufficient electrolyte intake	Recovery issues	YSI 3200 Conductivity Meter	Conductivity Sensor	Urine sample	Koh et al. (2016)
	Osmolality	100–1,200 mOsm/kg	Dehydration	Hyponatremia	Performance loss	Advanced Instruments Osmometer	Osmometer	Urine sample	Alizadeh et al. (2018)
	Osmolality	270–300 mOsm/kg	Dry eye syndrome, discomfort	Blurry vision, irritation	Decreased focus, eye strain	TearLab Osmolarity System	Osmometer	Eye (tear sample)	Bron et al. (2007)
Tears	Electrolytes	-	Excessive tear saltiness	Electrolyte imbalance in the eye	Eye strain, dry eye issues	Wearable Tear Electrolyte Sensor	Electrochemical Sensor	Eye (tear sample)	Yeo et al. (2015)
	Cortisol	∼10 ng/mL	Stress and fatigue	Recovery insufficiency	Increased mental stress, poor recovery	Tear ELISA Kits	Immunoassay Sensor	Eye (tear sample)	Iqbal et al. (2023)

Wearable biosensors designed for body fluid analysis must not only identify key physiological parameters but also exhibit high analytical performance. Sensitivity, selectivity, and the limit of detection (LOD) are critical indicators of precision, stability, and clinical applicability in such systems. Among these, LOD plays a decisive role in determining whether a sensor can reliably detect the typically low concentrations of biomarkers found in sweat, tears, or interstitial fluid. A lower LOD enables the detection of subtle physiological changes and early deviations from normal, which is essential for timely health monitoring and intervention. Accordingly, recent research has focused on improving LOD through advanced materials and optimized transduction mechanisms. Nanostructured electrodes, high-surface-area nanomaterials, and signal amplification strategies have been widely adopted to enhance sensitivity, achieving nanomolar or even sub-nanomolar detection limits for various biomarkers. Table 2 summarizes representative wearable sensor technologies and their reported LODs, highlighting how different sensing platforms—electrochemical, optical, and field-effect transistor (FET)-based—compare in terms of sensitivity.

**TABLE 2:** Wearable sensor technologies and their reported LODs for body fluids sensors.

Analyte (fluid)	Sensor type	Reported LOD	Source
Glucose (sweat)	Porous graphene electrochemical sensor (non-enzymatic)	<0.3 µM	Gao et al. (2023), Jamshidnejad-Tosaramandani et al. (2025)
Lactate (sweat)	MIP-based Ag nanowire electrochemical sensor	0.22 µM	Gao et al. (2023)
Cortisol (sweat)	Aptamer-GFET (extended-gate FET)	0.2 nM	Wang et al. (2022), Sheibani et al. (2021), Nguyen et al. (2025)
Cortisol (sweat)	Immuno-graphene FET (antibody-based)	0.0005 nM (500 fM)	Kim et al. (2025a)
Glucose (tears)	Plasmonic contact lens sensor (optical)	≈1 µM	Luo et al. (2025)
Glucose (ISF)	Microneedle enzymatic sensor (electrochemical)	8.65 µM	Luo et al. (2025)
L-Cysteine (sweat)	Graphene FET (flexible, transparent)	22 nM	Gao et al. (2023)
L-Cysteine (tears)	Graphene FET (flexible, transparent)	43 nM	Gao et al. (2023)

## 3 Sensors used for blood

### 3.1 Characteristics and parameters

Wearable sensors have transformed the monitoring of blood-related parameters in sports and healthcare through non-invasive technologies. Photoplethysmography (PPG) sensors are widely used to measure heart rate, heart rate variability (HRV), blood oxygen saturation (SpO2), and blood volume changes by analyzing light absorption in blood vessels (Seçkin et al., 2023; Yun et al., 2021). Optical sensors enhance these capabilities, enabling the measurement of blood oxygen levels and showing potential for non-invasive glucose monitoring (Quan et al., 2021). Electrical bioimpedance sensors track blood flow, volume, and hydration levels by assessing the body’s electrical resistance, while near-infrared spectroscopy (NIRS) sensors provide real-time insights into muscle oxygenation and tissue health (Ismail et al., 2023). Skin-interfaced microfluidic devices analyze sweat to estimate blood-related metabolites such as lactate and glucose, particularly valuable during physical activity (Iqbal et al., 2024). Among these, electrochemical sensors stand out for their versatility and precision. By measuring lactate, glucose, pH, and electrolytes in sweat, they offer a powerful tool for real-time metabolic and physiological monitoring, making them an essential component of wearable sensor technology for personalized health and performance optimization (Mao et al., 2023).

Blood serves as a primary diagnostic medium due to its complex biochemical composition. It maintains homeostasis by delivering oxygen and nutrients to tissues and removing metabolic waste. Key parameters include pH (7.35–7.45), osmolality (∼290 mOsm/kg), glucose levels (70–140 mg/dL), lactate concentration, and electrolyte balance. Deviations in these values can indicate metabolic disorders, dehydration, or physiological stress (Heikenfeld et al., 2018). Glucose monitoring is particularly critical for diabetic athletes, as it affects energy availability and performance. Similarly, blood lactate serves as an indicator of anaerobic threshold and recovery status (Jonathan et al., 2023).

### 3.2 Sensor technologies

**FIGURE 2:**
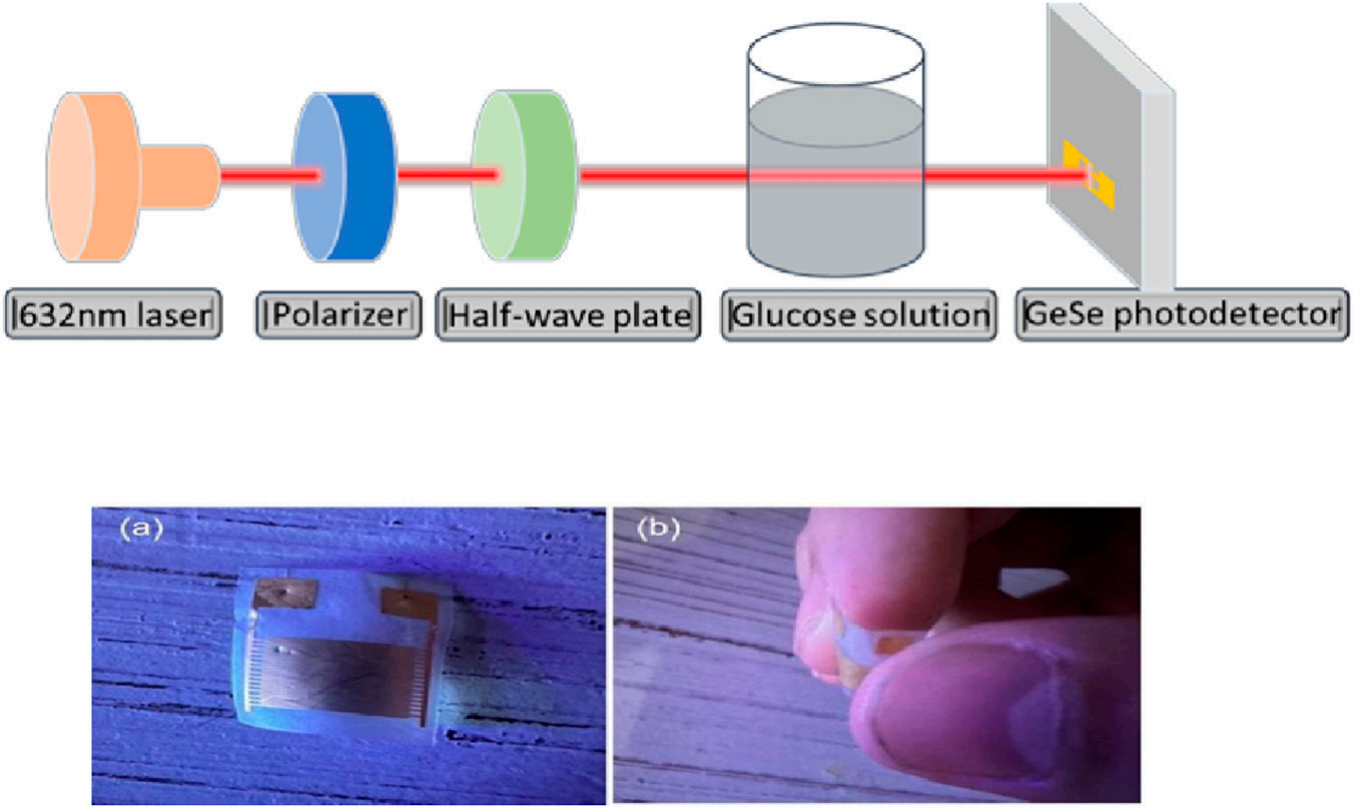
Representation of a non-invasive optical glucose-sensing system that measures blood glucose levels through light-polarization techniques enhanced with machine-learning algorithms for improved accuracy and user experience (Marco, 2024). **(a)** Illustration of the GeSe-based nanometric sensor architecture. **(b)** Demonstration of the sensor’s mechanical flexibility, showing that it can be freely bent without performance degradation.

The complexity of blood analysis has historically required laboratory-based methods; however, recent advances have brought about portable and wearable devices. Flexible nanomaterial sensors, such as those using GeSe, are integrated into wearable devices to measure blood glucose levels non-invasively (Figure 2). These sensors employ light polarization techniques and machine learning algorithms to enhance measurement accuracy, offering a seamless and reliable monitoring experience (Marco, 2024).

**FIGURE 3:**
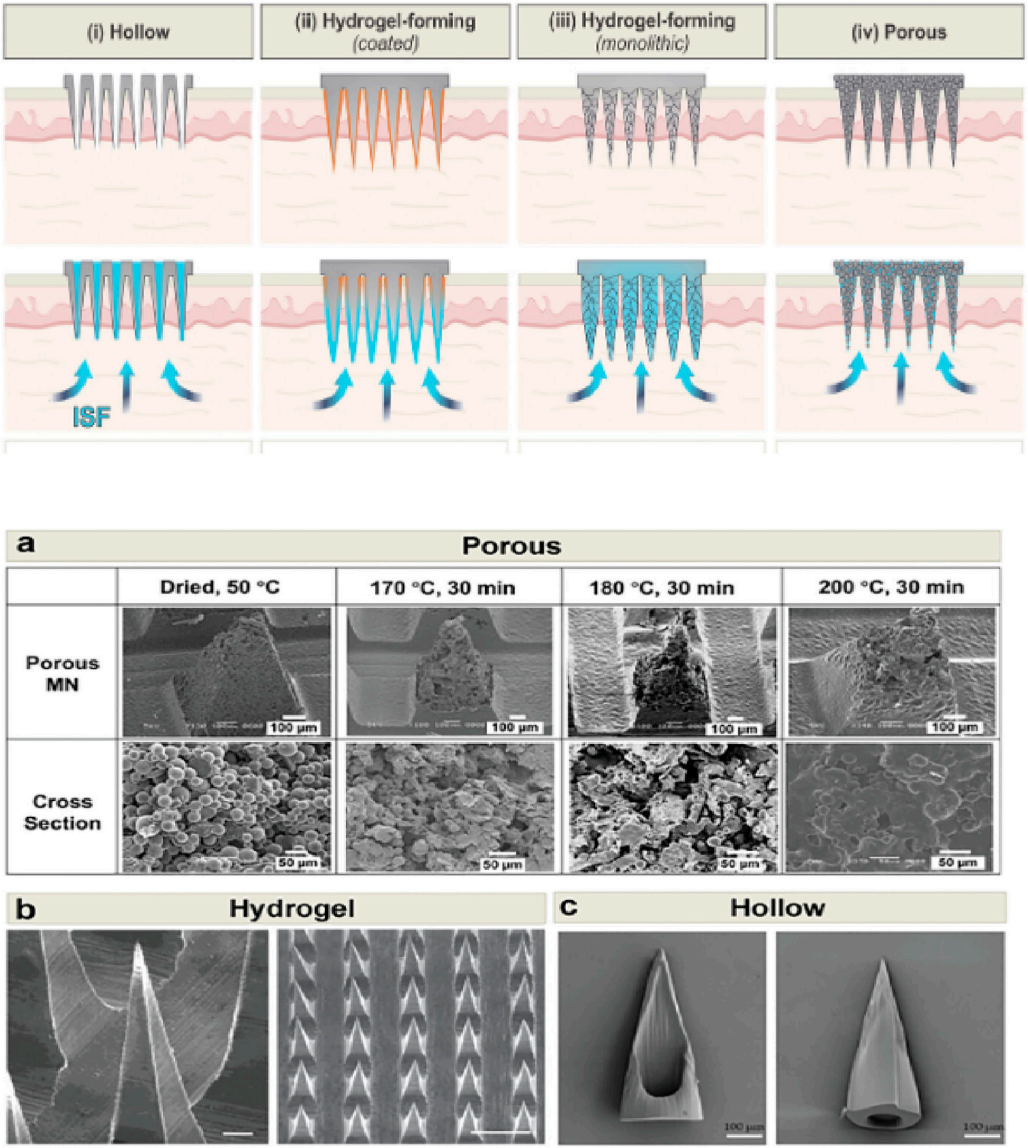
Detailed imagery of hydrogel-forming microneedle arrays combined with an electrochemical detector (the image above), highlighted by Scanning Electron Microscope (SEM) pictures of microneedles (the image below). This figure emphasizes the innovative approach to minimally invasive, real-time biochemical monitoring (Aroche et al., 2024). The SEM images include: **(a)** heat-treated porous PLA microneedles, **(b)** methacrylated hyaluronic acid (MeHA) hydrogel microneedles shown in top and side views, and **(c)** photolithographically fabricated hollow polymeric microneedles.

Electrochemical sensors are the gold standard for detecting analytes in blood due to their sensitivity, specificity, and adaptability to miniaturized designs. The i-STAT system, widely used in clinical settings, measures pH, electrolytes, and glucose but lacks the portability needed for wearable applications. Emerging wearable technologies aim to bridge this gap. Microneedle-based sensors (Figure 3) provide a minimally invasive solution for sampling interstitial fluid (ISF), which is compositionally similar to blood (Aroche et al., 2024). These sensors use polymeric microneedles combined with electrochemical detectors to measure lactate, glucose, and electrolytes in real time (Chua et al., 2013).

While ISF sensors with polymeric or hydrogel form microneedles and electrochemical detectors provide valuable insights into vital biomarkers, they still require skin penetration, which can be perceived as invasive and may cause discomfort or reluctance among users. This perception could impact user compliance. Furthermore, repeated application of these devices can lead to skin irritation or allergic reactions, and the variability in individual skin properties may affect the accuracy and reliability of the data collected. Additionally, the cost of these advanced sensors can be prohibitive, limiting accessibility, particularly in low-resource environments.

To enhance the appeal and utility of these technologies, there is a significant push towards improving and expanding non-invasive monitoring methods. Efforts are focused on refining sensor designs to eliminate skin penetration entirely, employing advanced algorithms and calibration techniques to adjust for individual differences, reducing manufacturing costs, and increasing educational outreach to inform potential users about the non-invasive options available. These strategies aim to make non-invasive health monitoring more acceptable, accurate, and accessible, thereby maximizing its potential in healthcare and sports science.

In addition, the incorporation of RFID technology enhances the functionality of wearable blood sensors. RFID modules allow real-time data transfer to mobile devices, enabling remote health monitoring. For instance, an RFID-integrated glucose sensor can continuously monitor and transmit blood glucose levels to smartphones, offering significant advantages for diabetic athletes (Sheng et al., 2022). While RFID offers passive operation, emerging BLE and Zigbee systems enable multi-node body networks for blood parameter telemetry, expanding connectivity and data throughput for real-time physiological monitoring.

### 3.3 Manufacturing approaches

Advancements in additive manufacturing have revolutionized the production of blood sensors. Techniques such as two-photon polymerization and stereolithography enable the fabrication of microstructures with sub-micrometer precision. These methods are particularly useful for creating microneedle arrays and sensor electrodes (Figure 3). Materials like polydimethylsiloxane (PDMS) and hydrogels are commonly used as substrates due to their biocompatibility and flexibility. Conductive materials, including graphene and silver nanowires, are integrated to enhance sensor performance. For example, graphene-coated microneedles exhibit superior electrical conductivity, making them ideal for electrochemical sensing (Yang et al., 2020). Hybrid manufacturing approaches are also gaining traction. Roll-to-roll printing is used to produce large-scale RFID antenna systems, which are integrated with 3D-printed sensor components to create compact, multifunctional devices. These methods reduce production costs while maintaining high performance and reliability.

In addition, the real-time monitoring of blood parameters has applications beyond athletic performance. For instance, in critical care, wearable blood sensors can track acid-base balance and electrolyte levels, reducing the need for frequent venipuncture. Additionally, in diabetes management, continuous glucose monitors (CGMs) improve glycemic control, reducing the risk of long-term complications. Lactate sensors are also used in clinical settings to assess sepsis severity and in sports science to optimize training regimens (Deulkar et al., 2024).

### 3.4 Hybrid and on-hand sensing platforms (lab-on-a-glove systems)

Recent “lab-on-a-glove” systems combine tactile sampling with on-site chemical sensing (UC San Diego Jacobs School of Engineering, 2017). Enzyme-coated electrodes on the fingertips can detect hazardous agents such as organophosphates or opioids upon surface contact (Mishra et al., 2017). Sweat-based glove sensors further enable in-motion monitoring of lactate and electrolytes during exercise (Luo et al., 2018). These glove-integrated biosensors bridge physical interaction and biochemical analytics—transforming the hand into an intuitive diagnostic interface.

## 4 Sweat sensors

### 4.1 Characteristics and parameters

Sweat is one of the most extensively studied biofluids in wearable sensor research due to its easy accessibility and non-invasive collection, serving as a valuable proxy for assessing both metabolic and hydration status (Meng et al., 2024; Zhang et al., 2024). This makes it particularly useful for monitoring athletic performance and general health. Sweat primarily consists of water, electrolytes (Na^+^, K^+^, Cl^−^), metabolites such as lactate and glucose, and trace biomarkers like cortisol (Koh et al., 2016; Sonner et al., 2015). Its pH typically ranges from 4.0 to 6.8, and deviations from this range may reflect skin-barrier dysfunction or metabolic stress (Choi and Fluhr, 2024); elevated pH levels often result from dehydration or excessive sweating, whereas acidic conditions are associated with irritation and inflammation. Electrolyte concentrations, especially sodium and potassium, provide insights into hydration and thermoregulation, while sweat conductivity reflects total ionic strength and thus overall electrolyte loss. Lactate concentration correlates with the anaerobic threshold and metabolic efficiency, marking exercise intensity (Hiroki et al., 2023). Owing to this dynamic composition, sweat is an excellent medium for real-time monitoring through wearable technologies, particularly in sports and occupational health applications. Notably, sweat composition varies by anatomical region—axillary sweat contains more lipids and volatile organic compounds, forehead sweat primarily reflects thermoregulation, and scalp sweat is linked to sebaceous activity—necessitating region-specific sensor calibration and substrate design (Gallagher et al., 2008; Baker, 2019).

### 4.2 Sweat sensor innovations

**FIGURE 4:**
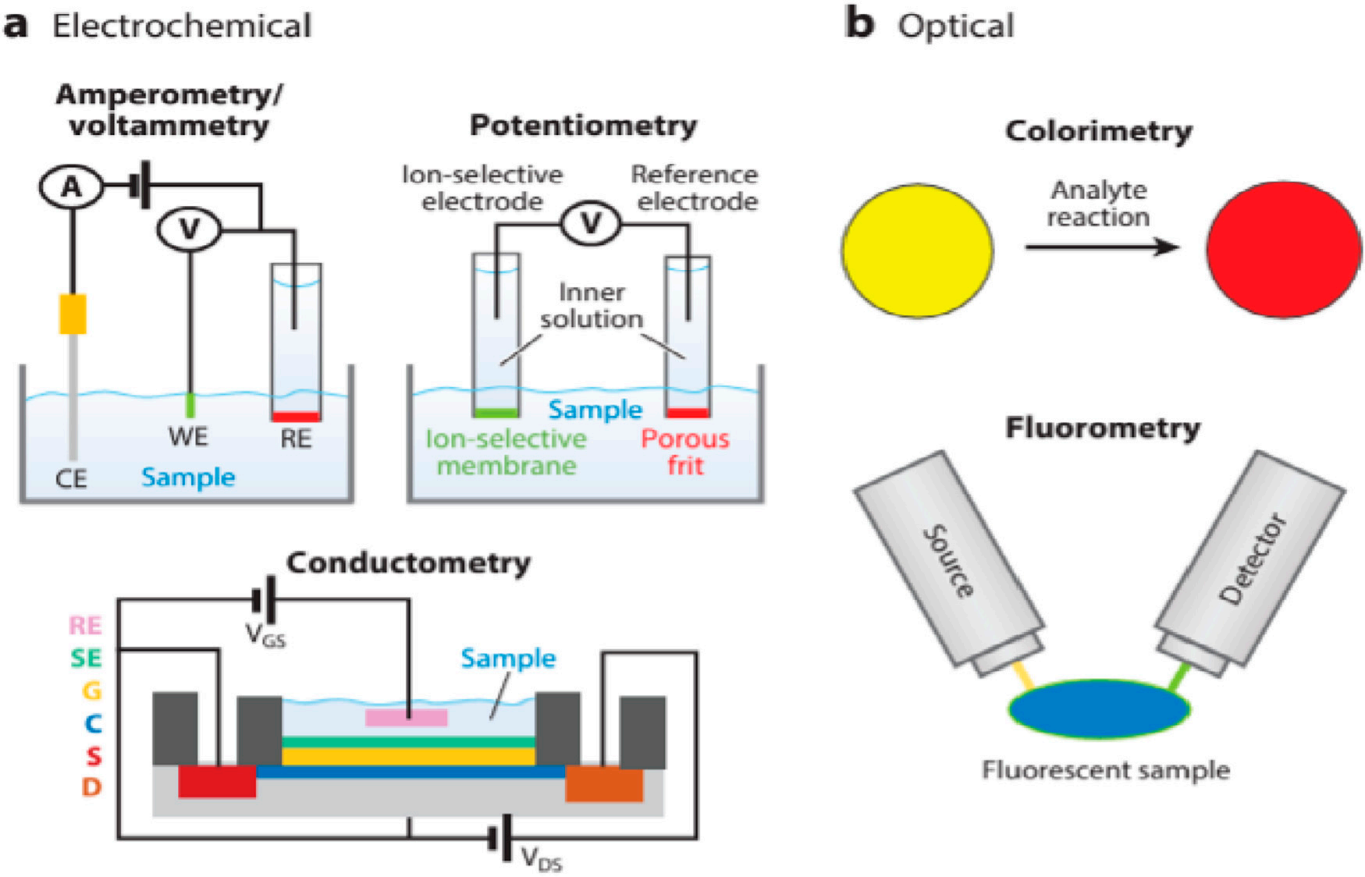
Illustration of the primary working mechanisms of sweat sensors, focusing on the electrochemical and optical detection methods. This figure highlights the sensors’ capabilities to analyze sweat for health monitoring, reflecting advancements in wearable technology (**(a)** Electrochemical, **(b)** Optical) (Bandodkar et al., 2019).

The working mechanism of these sensors typically involves the integration of microfluidic systems and biorecognition elements on flexible substrates. Sweat is directed through microfluidic channels to designated sensing regions, where selective detection occurs using electrochemical, optical, or colorimetric methods (Figure 4) (Bandodkar et al., 2019). For instance, enzymatic electrodes in the sensor oxidize glucose or lactate in sweat, generating an electrical signal proportional to the analyte concentration. These signals are then processed by embedded electronics and transmitted wirelessly for real-time monitoring. The integration of nanomaterials, such as graphene and metal-organic frameworks, enhances sensitivity and specificity. Sweat sensors offer a non-intrusive, continuous, and real-time monitoring approach, making them highly applicable for personalized healthcare, sports performance tracking, and disease management.

Recent advancements in sensor technology have driven the development of innovative devices for sweat monitoring, utilizing a variety of materials and techniques to enhance reliability, accuracy, and usability in real-time applications.

Metal oxide-based sensors, such as those combining RuO_2_ and TiO_2_, are renowned for their stability and ease of fabrication. Constructed using thick-film technology and optimized with an 80:20 ratio of RuO_2_ to TiO_2_, these sensors deliver excellent performance for sweat pH monitoring, fine-tuned through impedance spectroscopy (Pagar et al., 2024). Potentiometric sensors employ ion-selective electrodes (ISEs) crafted with advanced printing methods like aerosol-jet printing. These compact and cost-effective sensors feature pH-sensitive membranes on flexible substrates like TPU and PET, achieving reliable readings with a potentiometric response of −53.48 mV/pH on PET substrates (Dominiczak et al., 2024). Optical sensors provide a non-invasive solution by leveraging single-walled carbon nanotubes (SWCNTs) that emit near-infrared light for rapid, precise pH detection, particularly useful in healthcare and environmental monitoring (Sultana et al., 2023). Similarly, fiber-optic sensors, featuring tapered interferometers coated with pH-sensitive membranes, detect changes through wavelength shifts, offering broad-range sensitivity for physiological applications (Lei et al., 2023).

Flexible sensors are transforming sweat monitoring by conforming to the skin’s surface. Thin films made from Sb_2_O_3_/Sb offer lightweight, sensitive, and accurate readings across a wide pH range, while MoS_2_-polyaniline-based sensors provide stable, non-invasive monitoring for both natural and artificial sweat samples (Ghadge et al., 2024). Textile-based and colorimetric sensors further enhance practicality by integrating sweat monitoring directly into clothing. Curcumin-based fibers visually indicate pH changes through color variation, while biodegradable wool-based sensors like “Woolitmus” offer an eco-friendly, zero-waste alternative (Ghadge et al., 2024). Advanced wearable devices now combine microfluidic systems with electrochemical sensors to provide continuous analysis of sweat biomarkers. Systems like the Gatorade GX Patch direct sweat into sensing chambers using capillary action, measuring pH, electrolytes, and glucose levels. Flexible electronics using materials such as graphene and silver nanowires further enhance these devices by enabling wireless data transmission via Bluetooth or RFID. RFID-enabled sensors, particularly those integrated with microfluidic systems, are gaining popularity for their cost-effective real-time tracking of sweat pH, especially during physical activities, making them invaluable for athletes and individuals engaging in exercise (Ghadge et al., 2024).

Sweat cortisol sensors are another groundbreaking innovation, offering a non-invasive method for assessing stress and recovery. By using graphene oxide functionalized with antibodies or aptamers, these sensors achieve high specificity and sensitivity, enabling real-time stress monitoring (Riente et al., 2023). These advancements in sweat sensor technology are revolutionizing health and performance monitoring, providing precise and real-time insights with wide-ranging applications in sports, healthcare, and beyond.

### 4.3 Material and manufacturing insights for sweat sensors

Sweat sensors require biocompatible, flexible, and durable materials to withstand prolonged wear and mechanical stress during physical activities. Substrates such as polydimethylsiloxane (PDMS), thermoplastic polyurethane (TPU), and polyethylene terephthalate (PET) are commonly used for their desirable properties (Ates et al., 2022). Conductive materials like graphene, carbon nanotubes, and silver nanowires are integrated into electrodes to enhance electrical conductivity and sensitivity. For example, graphene-based materials are particularly suited for electrochemical sensing due to their high surface area and exceptional conductivity (Li et al., 2018). Additive manufacturing techniques, including fused deposition modeling (FDM) and stereolithography (SLA), have revolutionized the rapid prototyping of complex microfluidic systems. FDM is utilized for fabricating sweat channels and reservoirs, while SLA enables the creation of high-resolution sensing structures. Additionally, hybrid printing approaches, such as combining direct ink writing (DIW) of conductive inks with 3D-printed substrates, are increasingly employed to integrate sensing and communication modules (Gao et al., 2021). In literature, additive manufacturing, particularly the “one-step” fabrication process, has emerged as a key technique for developing flexible sensors. This method integrates layers of electrospun PVDF, printed silver electrodes, and electrospun TPU encapsulation to produce breathable, sweatproof, and highly responsive sensors ideal for wearable applications (Zhiqiang et al., 2024). Similarly, hydrothermal and one-pot preparation methods are utilized to create non-enzymatic electrochemical glucose sensors by functionalizing gold nanoparticles on aminated multi-walled carbon nanotubes, which are then crosslinked with carboxylated styrene-butadiene rubber and PEDOT: PSS. These flexible sensors, integrated onto screen-printed electrodes, enable high-sensitivity continuous glucose monitoring in sweat (Yuhua et al., 2024). The stamping-vacuum filtration dry transfer (SVFDT) technique offers a cost-effective approach for constructing wearable sweat glucose sensors. By combining a PVC stamp and vacuum-filtration, multi-walled carbon nanotube/PDMS film electrodes are fabricated, improving conductivity and stability for real-time sweat glucose detection (Youyuan et al., 2023). Another innovative approach, electro-assisted impregnation core-spinning technology (EAICST), is used to develop textile-based sweat sensors. This method combines impregnation coating and electrospinning to produce sheath-core electrochemical sensing yarns that are integrated into fabrics, enabling scalable production of durable, high-sensitivity, and washable sensors (Xiangda et al., 2024). Microfluidic and colorimetric analysis methods further advance wearable sensors, with hydrophilic yarn-based patches designed for sweat collection and real-time analysis through colorimetric indicators. Additionally, Janus nanofiber membranes improve directional sweat transfer, enhancing comfort and accuracy by preventing sweat accumulation (Wenze et al., 2023; Xuecui et al., 2024). Advanced materials and techniques such as 3D graphene foam and PDMS-based sensors have also demonstrated high sensitivity and stability in detecting uric acid in sweat, while waveguide and all-printed flexible sensors present innovative solutions for glucose and sweat monitoring (Mingrui et al., 2023; Balasubramanian et al., 2024; Chandradas et al., 2024). Together, these methods highlight the potential of wearable sweat sensors in healthcare, sports science, and beyond. To facilitate wireless data transfer, RFID technology is often incorporated into wearable sweat sensors, enabling remote monitoring by transmitting real-time data to smartphones or cloud-based platforms. Moreover, Bluetooth Low Energy (BLE) modules are frequently used for high-resolution data transmission over short distances.

In addition, wearable sweat sensors have a broad range of applications, particularly in sports science, occupational health, and medical diagnostics. For athletes, sweat monitoring provides real-time feedback on hydration and electrolyte levels, helping prevent dehydration and heat-related illnesses (Belabbaci et al., 2025). In occupational health, these devices are used to monitor workers in high-temperature environments, ensuring safe hydration and electrolyte balance. Medical applications include monitoring metabolic conditions, such as cystic fibrosis, through chloride concentration analysis. Recent studies have also explored the use of sweat sensors for early detection of infectious diseases, leveraging sweat biomarkers like cytokines and immune response markers (Ray et al., 2021).

### 4.4 Smart wound dressings and infection-responsive sweat sensors

Smart wound dressings now extend sweat sensing toward infection detection (Wang X. et al., 2024; Youssef et al., 2023). Changes in wound pH or bacterial metabolites such as pyocyanin signal infection onset, providing early indicators of bacterial colonization and inflammation (Wang X. et al., 2024; Gu et al., 2025). Aptamer-based hydrogels can even recognize pathogens such as *Staphylococcus aureus* or bacterial endotoxins, enabling selective and rapid detection in complex wound environments (Dsouza et al., 2022; Cristea et al., 2025). Some emerging “theranostic” systems not only detect infection but also release antibiotics or antimicrobial agents upon bacterial trigger, achieving on-demand therapy (Su et al., 2024; Cristea et al., 2025; Cao et al., 2023). This convergence of sweat and wound analytics redefines biosensing as both diagnostic and therapeutic, integrating real-time monitoring with intelligent drug delivery for advanced wound management (Yang et al., 2023; Vo and Trinh, 2025; Moradifar et al., 2024).

## 5 Saliva sensors

### 5.1 Characteristics and parameters

Saliva is increasingly recognized as a valuable biofluid for health monitoring due to its non-invasive collection, ease of handling, and rich biochemical composition. Secreted by salivary glands, it is primarily composed of water (approximately 99%) along with electrolytes, enzymes, hormones, glucose, and immune markers. Saliva acts as a mirror of systemic health, with many of its constituents closely correlating with blood levels (Lakshminrusimhan et al., 2024). Among the key parameters, pH typically ranges from 6.2 to 7.6 and serves as an indicator of oral health and systemic acid-based balance. A decrease in pH may signal oral infections or heightened acidity due to poor hydration, whereas an increase could point to reduced saliva flow or conditions like Sjögren’s syndrome. Cortisol, a stress biomarker, provides insights into hypothalamic-pituitary-adrenal (HPA) axis activity; elevated levels suggest acute stress, while chronic elevations may indicate long-term physiological strain or insufficient recovery (Choi et al., 2021). Salivary glucose emerges as a non-invasive metric for monitoring blood glucose levels in diabetic patients, offering a practical alternative to invasive blood sampling, especially for continuous monitoring. Additionally, immunoglobulin A (IgA), a critical antibody in mucosal immunity, reflects immune status. Low levels of salivary IgA are linked to a higher risk of infections, making it a significant parameter for athletes undergoing intense training (Ryland et al., 2022).

### 5.2 Wearable saliva sensors

Saliva sensors operate by detecting and analyzing biomarkers present in saliva, providing valuable insights into an individual’s health status (Anchidin-Norocel et al., 2024). These sensors typically utilize biochemical recognition elements, such as enzymes or antibodies, to specifically bind target analytes like glucose, lactate, or cortisol. Upon binding, a transducer converts this biochemical interaction into a measurable signal, often electrical or optical, which correlates with the concentration of the analyte. This non-invasive monitoring approach facilitates real-time health assessments, offering a convenient alternative to traditional methods.

**FIGURE 5:**
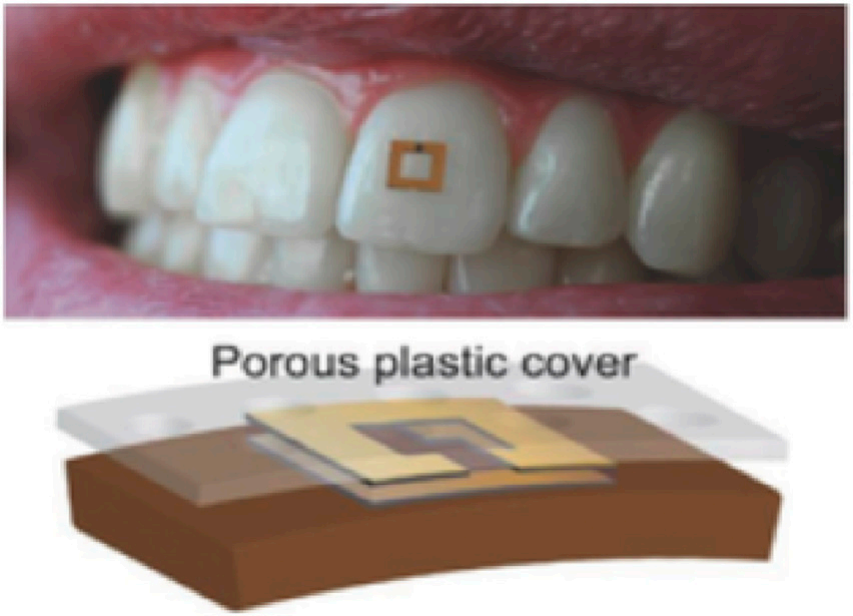
An RFID-based saliva sensor mounted on a tooth, illustrating the sensor’s functionality to detect changes in glucose levels by RF responses. This innovative approach enables real-time, non-invasive monitoring of stress and recovery in sports science (Tseng et al., 2018).

Wearable saliva sensors represent a transformative advancement in healthcare and performance monitoring, leveraging flexible electronics and advanced materials to provide real-time, non-invasive analysis with high sensitivity. Graphene-based sensors are particularly prominent in this field due to their superior conductivity, mechanical flexibility, and biocompatibility. For instance, salivary cortisol detection is achieved using graphene oxide electrodes functionalized with aptamers or antibodies, ensuring specificity and selectivity (Zaryab et al., 2024). Disposable biosensors, such as inkjet-printed devices utilizing conductive inks on biocompatible substrates like polyethylene terephthalate (PET), offer a cost-effective solution for saliva analysis. These single-use sensors deliver rapid and accurate measurements of biomarkers, including glucose and immunoglobulin A (IgA) (Ray et al., 2021). Additionally, RFID technology has been integrated into wearable saliva sensors to enable wireless data transmission. RFID based sensor, which senses glucose level changing by RF responses, is shown in Figure 5. Thus, RFID chips can transmit data to a smartphone, providing real-time stress assessments. Such innovations are particularly advantageous in sports science, where timely feedback on stress and recovery is essential (Choi et al., 2021).

### 5.3 Insights into materials and manufacturing processes for saliva sensors

The development of saliva sensors necessitates materials capable of withstanding the wet and enzymatically active environment of the oral cavity. Hydrogels are often utilized as substrates due to their biocompatibility and ability to retain moisture. To enhance sensor performance, conductive materials such as carbon nanotubes (CNTs) and silver nanowires are integrated into these substrates. Additive manufacturing techniques, including stereolithography (SLA) and direct ink writing (DIW), have proven essential in the fabrication of saliva sensors, particularly for creating intricate microfluidic channels that direct saliva samples to specific sensing regions, thereby improving measurement accuracy. Inkjet printing is especially effective for depositing conductive materials, facilitating the production of lightweight, flexible, and disposable saliva sensors. In the literature, 3D printing is being utilized to fabricate flexible ion-selective field effect transistors (ISFETs) for saliva analysis (Chao et al., 2019). This technique involves the hybridization of printed organic ion-selective electrodes with inorganic transistors, providing high sensitivity and selectivity in detecting ions such as ammonium, potassium, and calcium, as reported by Bao et al. (2019). Another innovative approach is the Spin Coating and Plasma Treatment, part of the Spin Coating-Plasma treatment-Coprecipitation (SPC) strategy (Tingjun et al., 2024). This method is used to develop super-hydrophilic gel sensors, significantly enhancing the sensor’s surface properties to improve its effectiveness in saliva glucose monitoring, according to Chen et al. (2024). Additionally, Dip-Coating is a straightforward and efficient manufacturing technique for creating liquid flexible sensors. It involves coating a cellulose fabric with a conductive composite material, making it a cost-effective option suitable for large-scale production, as described by Siyi and Yinxiang (2019) (Bi and Lyu, 2019). Each of these techniques contributes uniquely to advancing sensor technology, offering tailored solutions for specific biochemical analyses. Additionally, the integration of communication modules, such as RFID and near-field communication (NFC) systems, allows for wireless data transfer. These modules are often fabricated using roll-to-roll printing techniques, enabling large-scale production while ensuring the sensors remain reliable and high-performing.

Furthermore, wearable saliva sensors have diverse applications, ranging from stress and hydration monitoring in athletes to chronic disease management. In sports science, salivary cortisol and IgA levels are used to assess recovery and immune function, guiding training regimens to minimize overtraining risks (Ryland et al., 2022). In medical diagnostics, saliva sensors are being developed for early detection of conditions such as diabetes and oral cancers. Glucose monitoring devices offer a non-invasive alternative for diabetic patients, while salivary biomarkers for oral cancers are being explored for rapid, point-of-care diagnostics.

### 5.4 Smart pacifier biosensors for neonatal monitoring

In neonatal care, saliva-based “smart pacifiers” replace invasive blood draws (Lim et al., 2022; García-Carmona et al., 2019). These pacifiers integrate microfluidic channels and ion-selective electrodes to continuously monitor sodium and potassium levels (Lim et al., 2022). Data transmitted via Bluetooth enables early detection of dehydration or kidney stress. Other prototypes target glucose monitoring in neonatal hypoglycemia (García-Carmona et al., 2019; Zhou et al., 2024). Such soft, baby-safe devices highlight how biosensing can be empathetic, non-invasive, and child-centered.

## 6 Urine sensors

### 6.1 Characteristics and parameters

Urine, a biofluid excreted by the kidneys, serves as an accessible and rich diagnostic medium, providing valuable insights into the body’s hydration status, electrolyte balance, and metabolic health (Gao et al., 2024; Lee et al., 2024). Unlike blood, urine analysis captures cumulative physiological changes over time, making it particularly useful for long-term monitoring. Key diagnostic parameters include pH, osmolality, electrolytes, proteins, glucose, and ketones. The pH of urine, typically ranging from 4.5 to 8.0, reflects the body’s acid-based balance, with alkaline urine potentially indicating urinary tract infections (UTIs) or dietary influences, while acidic urine is often linked to high protein intake or conditions like ketoacidosis. Urine osmolality, generally between 100 and 1,200 mOsm/kg, directly measures solute concentration and hydration status, with high values often signaling dehydration or kidney stress, and low values suggesting overhydration or renal dysfunction. Electrolyte levels, including sodium, potassium, and chloride, are critical for muscle function and physiological stability. Proteinuria, the presence of excess proteins in urine, is a marker of kidney dysfunction or systemic inflammation. Additionally, glucose and ketones are significant markers in diabetes management, indicating hyperglycemia or ketosis (Shirreffs and Maughan, 1998; Li et al., 2018). Its composition and variability, urine is an excellent candidate for wearable monitoring systems, particularly for athletes and patients with chronic conditions.

### 6.2 Urine sensor development

Wearable urine sensors are rapidly emerging as innovative and practical tools for non-invasive health monitoring, offering significant advancements in real-time health tracking (Fakhr et al., 2024). These sensors typically utilize electrochemical, optical, or piezoelectric principles to transduce chemical interactions into measurable electrical signals. These devices aim to deliver continuous data on hydration status, electrolyte balance, and metabolic health, eliminating the need for frequent laboratory visits and traditional invasive procedures. They are particularly beneficial in medical diagnostics and for individuals such as athletes or workers in physically demanding environments, who require consistent monitoring of their physiological status.

One of the key technologies driving this innovation is flexible electrochemical sensors, which are engineered to detect parameters like pH, osmolality, and electrolyte concentrations. These sensors often utilize screen-printed electrodes coated with conductive polymers such as poly (3,4-ethylenedioxythiophene) (PEDOT), a material renowned for its exceptional sensitivity and durability (Gao et al., 2021). In this context, flexible and wearable humidity sensors, which are used as urine sensors and manufactured with PEDOT: PSS, provide an effective solution for detecting urinary incontinence. These sensors can be integrated between the cover stock and the acquisition/distribution layer (ADL) of the adult diaper (Tekcin et al., 2022). This placement allows the sensor to effectively detect urinary incontinence while preventing direct contact with the skin. The use of such advanced materials ensures accurate and reliable performance, even in challenging conditions.

Another essential component of wearable urine sensors is the integration of microfluidic platforms, which streamline the sample collection process. These platforms leverage capillary forces to direct urine samples to the sensing regions passively, requiring minimal user intervention. This design is particularly advantageous for continuous monitoring, as it reduces the burden on users while maintaining consistent data collection. Such systems have proven valuable for monitoring individuals exposed to high-stress environments, including athletes and emergency responders (Yang et al., 2020).

In recent advancements, optical and multi-parameter sensing technologies have gained prominence. These hybrid sensors combine optical and electrochemical methods to measure multiple biomarkers simultaneously, such as pH, glucose, and ketones. This multifunctional capability provides a holistic view of an individual’s health, enhancing the potential for early detection of conditions like dehydration, metabolic disorders, or stress-related imbalances.

Collectively, these advancements in wearable urine sensor technology represent a paradigm shift in health monitoring, combining comfort, accessibility, and precision to empower individuals and healthcare professionals alike. As research continues to refine these systems, their potential applications are poised to expand, driving transformative impacts in personalized medicine and public health.

### 6.3 Advances in material and manufacturing for urine sensors

The materials used in wearable urine sensors must withstand the corrosive nature of urine while ensuring accurate biomarker detection (Anshin et al., 2024). Hydrophobic polymers like polyethylene terephthalate (PET) are often utilized for device housings due to their durability, while conductive materials such as silver nanowires and graphene are incorporated into sensing elements to enhance performance. Various manufacturing techniques have been developed to enhance the design and functionality of urine sensors, each employing unique methods and materials. The Pad Printing Technique, as detailed by Tekçin and Bahadır (2024), uses silver and silver/nickel inks to fabricate flexible urine sensors (Meltem and Kursun, 2024). This process involves multiple sintering passes to improve conductivity and surface morphology, essential for accurately detecting changes in resistance when exposed to urine. Another innovative approach is the use of Electrospinning and Carbon Nanofibers, where phosphated lignin is incorporated to enhance the flexibility and thermal stability of the fibers. This green strategy, described by Zheng et al. (2022), increases the sensor’s ability to detect uric acid in urine through improved molecular interactions and electron transmission (Hao et al., 2022). Lastly, Contactless Jet Printing, as implemented by Zhang et al. (2023), offers a rapid manufacturing solution for creating ultrathin, highly sensitive moisture sensors (Lan et al., 2023). These sensors are directly printed onto diapers or undergarments, facilitating continuous urine monitoring while maintaining user comfort. Each technique represents a significant advancement in sensor technology, addressing specific challenges in moisture detection and wearable comfort.

Furthermore, wearable urine sensors are transforming the way hydration, electrolyte balance, and kidney function are monitored, offering significant benefits for athletes, patients with chronic illnesses, and individuals in high-risk professions. In sports science, these sensors provide real-time feedback on hydration and electrolyte levels, helping athletes prevent dehydration and heat-related illnesses. By monitoring osmolality and sodium excretion during high-intensity activities, these devices enable tailored hydration strategies to optimize performance (Shirreffs and Maughan, 1998). In medical diagnostics, wearable urine sensors are invaluable for patients with chronic kidney disease, facilitating continuous monitoring of renal function. Diabetic patients also benefit from these devices, as they enable glucose and ketone monitoring, reducing the need for invasive blood tests. In occupational health, wearable urine sensors are critical for ensuring proper hydration and electrolyte balance under extreme conditions, such as in mining or firefighting environments (Li et al., 2018). Additionally, researchers are exploring the potential of urine biomarkers for the early detection of urinary tract infections (UTIs) and systemic inflammatory conditions, using wearable sensors to provide rapid, and point-of-care diagnostics (Yang et al., 2020).

### 6.4 Diaper-integrated smart urine sensors

Smart diapers transform routine care into real-time health tracking (Tanweer et al., 2024). Printed impedance or capacitive sensors detect urination events and transmit data via Bluetooth (Xiong et al., 2024). Some systems measure uric acid or ionic composition to assess hydration and renal health (Shitanda et al., 2021). Commercial devices like Monit™ and Opro9™ already demonstrate this concept in infant and elderly care, helping to prevent rashes and monitor urinary infections (Opro9, 2025). Disposable yet intelligent, these systems redefine the interface between comfort and diagnostics.

## 7 Tears sensors

Tears have become a focal point for wearable sensor applications, recognized for their easy accessibility and the wealth of biomarkers they carry. Parameters like osmolality, electrolytes, and stress-related hormones such as cortisol make tears especially valuable for monitoring. These biomarkers yield crucial insights into not only ocular health but also broader systemic conditions, making tear analysis an impactful tool in sports, healthcare, and ophthalmology (Bron et al., 2007). For athletes, in particular, the ability to monitor changes in tear osmolality and cortisol levels offers direct benefits for managing hydration and stress, critical factors in achieving optimal performance. The use of advanced microfluidic and electrochemical technologies in wearable devices facilitates the real-time, non-invasive tracking of these biomarkers, significantly advancing personalized healthcare and sports science initiatives.

### 7.1 Characteristics and parameters

Tear fluid contains various biomarkers that provide critical insights into ocular and systemic health. One such marker is osmolality, a vital parameter for evaluating dry eye syndrome and systemic hydration. Typically ranging between 270 and 300 mOsm/kg, tear osmolality serves as an important indicator of ocular surface health, as highlighted in studies by (Bron et al., 2007). This measurement is widely utilized in both clinical and research settings to monitor changes in the tear film associated with environmental stressors or pathological conditions.

Another significant biomarker is cortisol, a hormone presents in tears at concentrations of approximately 10 ng/mL. Elevated levels of tear cortisol have been linked to increased stress and mental fatigue, making it a valuable, non-invasive tool for assessing physiological strain. Research by (Iqbal et al., 2023) emphasizes the potential of tear cortisol measurements to provide real-time insights into an individual’s stress response, offering applications in both healthcare and performance monitoring.

Lastly, the levels of electrolytes such as sodium and potassium play a crucial role in maintaining ocular homeostasis. These electrolytes not only support the integrity of the tear film but also serve as indicators of electrolyte imbalances that could impact both eye and systemic health. Yeo et al. (2015) underline their importance in diagnosing and managing conditions related to hydration and ionic disturbances. Together, these biomarkers—osmolality, cortisol, and electrolytes—represent a comprehensive approach to understanding and monitoring the intricate connections between ocular health and systemic wellbeing (Wang Z. et al., 2024).

### 7.2 Tear sensor applications

Tear sensors are becoming increasingly important tools for monitoring our health, particularly in areas like ocular health, hydration, and stress management (Rajan et al., 2024). For people with conditions such as dry eye syndrome, devices like the TearLab Osmolarity System use microfluidic technology to measure tear osmolality, helping with diagnosis and treatment (Zazoum et al., 2022). Similarly, wearable glasses with built-in optical sensors provide real-time updates on hydration levels by tracking changes in tear osmolality, making it easier to maintain eye health (Lan et al., 2023).

When it comes to managing stress, tear sensors offer unique insights by detecting cortisol levels, a key stress hormone. Electrochemical sensors equipped with specialized molecules like aptamers or antibodies are highly sensitive, making them ideal for tracking stress, especially for athletes (Ghadge et al., 2024). Some advanced versions even use RFID technology to send real-time data to smartphones, making stress management more accessible (Ungaro et al., 2015).

For those concerned about hydration and electrolyte balance, wearable patches with ion-selective electrodes (ISEs) can measure sodium and potassium levels in tears, helping to prevent imbalances that could affect overall health (Yeo et al., 2015). On the inflammation front, tear sensors break new ground by detecting inflammatory markers, offering a way to diagnose conditions like conjunctivitis or autoimmune diseases. Wearable sensors that analyze proteins in tears are also emerging, using techniques like mass spectrometry to provide a deeper understanding of both eye and systemic inflammation (Iqbal et al., 2023).

In addition, tear sensors are expanding into broader health applications, such as glucose monitoring for diabetes. Devices using advanced nanomaterials now offer a non-invasive way to track glucose levels continuously, improving the lives of those managing diabetes (Sandra, 2023; Zhou, 2024). Tear sensors built into contact lenses, using fluorescence technology, are another breakthrough. These can detect key biomarkers for inflammation and disease, enabling personalized and immediate care (Shi et al., 2024; Shi et al., 2023). With these advancements, tear sensors are proving to be more than just medical devices—they are tools that empower people to take charge of their health in a non-invasive, real-time way. Whether managing stress, hydration, or chronic diseases, these sensors are paving the way for a future of more personalized and accessible healthcare.

### 7.3 Materials and manufacturing for tear sensors

Tear sensors detect biochemical markers in tear fluid using advanced technologies such as electrochemical, optical, and piezoelectric mechanisms. These sensors frequently incorporate nanomaterials like graphene, carbon nanotubes, and metallic nanoparticles to achieve higher sensitivity and specificity (Rajan et al., 2024). For example, electrochemical tear sensors measure glucose levels for diabetes management by detecting the oxidation of glucose molecules through enzymatic reactions on the sensor’s surface. Optical sensors utilize fluorescence or changes in refractive index to detect analytes like lactate or electrolytes, which are crucial for assessing hydration and metabolic health. Piezoelectric mechanisms excel in measuring tear flow and viscosity, offering valuable insights into ocular surface disorders such as dry eye disease. When integrated into wearable devices like smart contact lenses, tear sensors provide continuous and non-invasive health monitoring, presenting a transformative approach to personalized healthcare.

The functionality of tear sensors relies on specialized materials and precise manufacturing techniques to accommodate the sensitive ocular environment. Materials such as polydimethylsiloxane (PDMS) deliver the flexibility and biocompatibility needed for wearable tear sensors, while graphene and silver nanowires enhance electrical sensitivity, making them ideal for electrochemical applications targeting tear analysis (J et al., 2023). Advanced manufacturing methods, such as 3D printing, enable the precise fabrication of microfluidic channels to optimize tear collection and analysis (Kim et al., 2018). Additionally, inkjet printing facilitates the production of disposable electrodes, reducing contamination risks in single-use tear sensors (Riente et al., 2023).

These technological advances have significant implications for sports and healthcare. In sports, real-time cortisol tracking through tear sensors supports stress management and recovery optimization for athletes. In healthcare, tear osmolality sensors are vital for diagnosing and monitoring dry eye syndrome, a condition particularly common among athletes frequently exposed to outdoor environments. Furthermore, analyzing tear electrolytes and proteins enables the early detection of systemic health issues, including autoimmune diseases and electrolyte imbalances, offering valuable insights into overall health management.

### 7.4 Ring-based multimodal platforms for tear and skin biomarkers

Beyond ocular applications, the integration of tear sensing with multimodal wearable platforms such as smart rings bridges biochemical and physiological analytics, enabling holistic athlete monitoring. Smart rings provide continuous, non-invasive insight into physiological trends such as heart rate, sleep, and temperature (Fiore et al., 2024). Advanced prototypes integrate optical and electrochemical sensors to estimate glucose and blood pressure non-invasively (Sempionatto and Wang, 2017; Tang et al., 2025). Compact, waterproof, and discreet, they extend biosensing to daily life—merging comfort, analytics, and personalized health intelligence.

## 8 Other body fluids

Wearable sensor technology is breaking new ground by incorporating less conventional biofluids, such as semen and vaginal fluids, to provide advanced insights into health monitoring, including applications tailored for athletes. These biofluids contain critical biomarkers that offer valuable information on hydration, recovery, and reproductive health, presenting new opportunities for optimizing athletic performance and wellbeing.

Semen serves as a biomarker-rich fluid that can reflect systemic health and fertility parameters. Advanced sensor systems are being developed to analyze semen’s pH (7.2–8.0), sperm motility, and electrolyte concentrations in real time. Though wearable solutions are in the research phase, portable microfluidic and electrochemical sensors are being optimized for non-invasive sperm motility assessments and monitoring of oxidative stress markers like prostate-specific antigen (PSA). These technologies, when adapted to athletes, could monitor hormonal or oxidative imbalances, which directly impact recovery and physical performance (Heikenfeld et al., 2018).

Similarly, reproductive health features unique markers such as a specific pH range (3.8–4.5) within the female reproductive tract. Advanced pH sensors integrated into smart tampons or patches enable non-invasive monitoring of conditions like bacterial vaginosis and yeast infections, as well as the assessment of inflammatory markers such as cytokines. These technologies are particularly useful for athletes, providing real-time insights into hydration levels, immune function, and overall reproductive health. This information is vital for managing the demands of intense physical activities and optimizing recovery periods. Additionally, the use of biosensors to monitor cervical mucus can help in tracking electrolyte shifts and hormonal changes, which can be critical for tailoring training regimens to individual physiological cycles (Choi et al., 2021).

Emerging RFID-enabled wearable systems further enhance these applications. Devices such as sanitary pads equipped with biosensors measure blood parameters like hemoglobin A1c and electrolyte levels, providing a new layer of non-invasive, continuous monitoring. These can be particularly valuable for female athletes in managing menstrual health and its impact on performance. Furthermore, the integration of IoT platforms and AI analytics allows for real-time decision-making, offering athletes actionable insights for tailored hydration, nutrition, and training adjustments.

These innovative sensors, by monitoring biofluids like semen and vaginal fluids, are paving the way for highly specialized health assessments in athletes. By leveraging advanced materials such as graphene and flexible hydrogel interfaces, these devices ensure biocompatibility and precision in dynamic conditions. Future iterations of these sensors, combined with miniaturized electronics and cloud-based analytics, are poised to revolutionize athlete health monitoring, enabling peak performance and reducing injury risks through personalized, data-driven interventions.

## 9 Advanced materials and manufacturing techniques

### 9.1 Materials for wearable sensors

Materials science plays a critical role in the design and functionality of wearable sensors for body fluid monitoring. The materials used in these devices must meet strict criteria, including biocompatibility, flexibility, durability, and responsiveness to physiological environments. Given that wearable sensors often operate under dynamic conditions, they require materials capable of maintaining performance despite mechanical stress and exposure to biofluids.

Flexible polymers and substrates, such as polydimethylsiloxane (PDMS), thermoplastic polyurethane (TPU), and polyethylene terephthalate (PET), are widely used in wearable sensors. PDMS stands out for its biocompatibility, optical transparency, and elasticity, making it ideal for skin-conforming applications. TPU, known for its excellent mechanical strength, is frequently employed in flexible microfluidic systems designed for analyzing sweat and urine (Atsani Susanto et al., 2023).

Advanced conductive materials, including graphene, carbon nanotubes (CNTs), and silver nanowires, significantly enhance the electrical performance of wearable sensors. Graphene, with its high surface area and exceptional conductivity, is commonly integrated into electrochemical sensors for detecting biomarkers like glucose and lactate. Silver nanowires, prized for their stretchability and conductivity, are especially suitable for flexible electrodes.

In terms of enhancing sensitivity and expanding detection limits, the limits of detection (LoDs) and sensitivity are crucial parameters that determine the efficacy of wearable sensors in health monitoring, particularly for detecting low-concentration biomarkers essential for early diagnosis and management in personalized medicine and chronic disease scenarios. Notably, graphene-based sensors, such as graphene-based field-effect transistors (GFETs), have achieved remarkably low LoDs—0.022 × 10^−6 M for L-cysteine in sweat and 0.043 × 10^−6 M in artificial tears—demonstrating their capability for sensitive biomarker detection across various body fluids (Huang et al., 2022). Additionally, electrochemical sensors have been refined to provide low LoDs by incorporating advanced materials and microfluidic integration, making them essential for tracking a broad spectrum of biomarkers in sports medicine and chronic illness management (Raymond et al., 2024). The development of dual biofluid sampling platforms has enabled simultaneous monitoring of sweat and interstitial fluid, offering comprehensive biomarker analysis with correlations closely aligned to blood levels, which is vital for extending the sensors’ clinical applicability (Kim et al., 2018).

Hydrogels are another critical component, valued for their ability to retain moisture and mimic the mechanical properties of biological tissues. They are particularly effective in saliva and urine sensors, where direct contact with biofluids is essential. Additionally, hydrogels embedded with ionic conductors or nanomaterials can function as active sensing elements, enhancing signal transduction (Sun Y. et al., 2024).

Smart materials, such as shape-memory alloys and piezoelectric polymers, are being increasingly integrated into wearable sensors to enable advanced functionalities. For example, piezoelectric materials can generate electrical signals in response to mechanical deformation, creating self-powered sensor systems that eliminate the need for external batteries (Gao et al., 2021).

### 9.2 Additive manufacturing in wearable sensor fabrication

Additive manufacturing, commonly known as 3D printing, has revolutionized the production of wearable sensors by enabling rapid prototyping, customization, and the integration of complex structures. Several advanced 3D printing techniques are utilized in wearable sensor development to meet the specific demands of different applications. Stereolithography (SLA), which employs photopolymerization, is ideal for creating high-resolution structures and is widely used to fabricate microfluidic components and sensor housings. This technique excels in producing saliva and urine sensors with intricate channel designs that efficiently direct biofluids to sensing regions (Li et al., 2018). Fused Deposition Modeling (FDM), a versatile and cost-effective method, is commonly used to create flexible substrates and durable housing for wearable sensors. Materials such as TPU and PETG are frequently employed in FDM to fabricate sweat sensors with complex microfluidic paths, ensuring efficient sweat collection and analysis. Direct Ink Writing (DIW) allows for the precise deposition of conductive inks and polymers onto flexible substrates, facilitating the creation of integrated sensor-electrode systems. This approach is particularly effective for hybrid sensors that combine electrochemical and optical sensing modalities (Ray et al., 2021). Two-Photon Polymerization (TPP) is another innovative technique that enables the fabrication of nanoscale structures with exceptional precision. It is widely used for creating microneedle arrays in blood sensors, allowing for minimally invasive sampling and enhanced sensitivity (Yang et al., 2020). Together, these techniques have significantly advanced the capabilities and versatility of wearable sensor technologies.

### 9.3 Integration of electronics

The integration of electronic components such as RFID and Bluetooth modules significantly enhances the functionality of wearable sensors by enabling wireless data transmission and real-time monitoring. RFID technology serves as a cornerstone for wearable sensors, particularly in healthcare and sports applications. RFID-enabled sensors facilitate remote monitoring by transmitting data wirelessly to smartphones or cloud platforms, with these modules often fabricated using roll-to-roll printing to ensure scalability and cost-efficiency (Gao et al., 2021). Similarly, Bluetooth Low Energy (BLE) modules are widely employed in wearable sensors for short-range, high-resolution data transmission. BLE is particularly suited for real-time monitoring during physical activity, offering continuous feedback on physiological parameters (Liu et al., 2021). Additionally, advances in flexible electronics have enabled the creation of wearable sensors that conform seamlessly to the skin. Flexible printed circuit boards (FPCBs) integrate sensors, processors, and communication modules into compact, durable devices. These circuits, fabricated with conductive inks on polymer substrates, combine flexibility with reliability, making them ideal for wearable applications (Li et al., 2018).

### 9.4 Hybrid manufacturing approaches

Hybrid manufacturing combines multiple fabrication techniques to optimize sensor performance and reduce production costs. For instance, 3D printing is often combined with roll-to-roll printing to fabricate wearable sensors with integrated communication modules. These approaches allow for the simultaneous production of structural and electronic components, streamlining the manufacturing process.

### 9.5 Applications and future directions

The use of advanced materials and manufacturing techniques has significantly expanded the applications of wearable sensors across healthcare, sports science, and occupational health. Future research should prioritize the development of sustainable materials, such as biodegradable polymers, to minimize environmental impact. Efforts should also focus on enhancing sensor sensitivity and selectivity through the integration of nanomaterials, enabling more precise and reliable monitoring. Additionally, scaling up manufacturing processes will be essential to ensure these technologies remain both affordable and accessible. The synergy of additive manufacturing and flexible electronics is poised to drive the next-generation of wearable sensors, enabling seamless integration into daily life and advancing personalized healthcare solutions. Future research should converge soft bioelectronics, AI-driven interpretability, and sustainable 3D manufacturing toward clinically standardized, ethically compliant, and globally deployable wearable platforms. In parallel with these technological advances, data privacy, ethical governance, and sustainability must form the backbone of global-scale biosensing deployment.

#### 9.5.1 Ethical, scalable, and secure data ecosystems

As wearable biosensors increasingly integrate with AI- and IoT-based health infrastructures, data privacy and ethical governance become pivotal. Beyond technical performance, sensor networks must comply with strict data protection frameworks such as the General Data Protection Regulation (GDPR) to ensure secure, anonymized data flow across interconnected systems. Recent frameworks emphasize GDPR-compliant data handling and federated learning models that preserve user privacy while enabling large-scale analytics (Zaguir et al., 2024).

Moreover, the long-term sustainability and scalability of IoT-enabled healthcare demand circular design principles—reducing electronic waste, optimizing energy use, and enabling device recyclability through modular manufacturing (Brauneck et al., 2023; Saha et al., 2025). Integrating these ethical, environmental, and economic considerations into next-generation biosensing platforms will be critical for achieving global deployment, clinical standardization, and equitable healthcare access.

### 9.6 Wireless data transmission technologies in wearable sensors

Modern wearable biosensors depend on seamless, low-power wireless communication. Among available options, Radio-Frequency Identification (RFID) remains the foundation for passive, battery-free data exchange (Kim C. et al., 2025). Yet, new standards—Near-Field Communication (NFC), Bluetooth Low Energy (BLE), and Zigbee—are extending range, bandwidth, and network flexibility (Sun et al., 2022a; Kong et al., 2024). Table 3 shows wireless technology used for body fluids sensors.

**TABLE 3:** Wireless technologies for wearable body fluid sensors.

Technology	Range	Power	Data rate	Main strengths/Applications	Source
RFID	∼5–20 cm (passive), up to 10 m (active)	Passive/Battery	Low–Moderate	Battery-free, low-cost patches for sweat/glucose sensing	Kim et al. (2025b), Sun et al. (2022b)
NFC	∼5–10 cm	Passive	Moderate (424 kbps)	Smartphone-compatible, secure, two-way data and power	Sun et al. (2022b), Lazaro et al. (2023)
BLE	∼10–30 m	Battery	∼1 Mbps	Real-time streaming in fitness bands, rings, and smart patches	Xu et al. (2024), Tipparaju et al. (2021)
Zigbee	∼10–100 m (mesh)	Battery	250 kbps	Multi-sensor networking for motion or rehabilitation systems	Xu et al. (2024)
Wi-Fi	∼50 m	Battery/Mains	>10 Mbps	High-data-rate medical monitors; energy-intensive	Sun et al. (2024b)

RFID and NFC: RFID enables wireless data transfer through electromagnetic coupling between a reader and tag. Passive RFID tags, powered directly by the reader field, are ideal for thin, battery-free patches that monitor biomarkers such as sweat pH or glucose. NFC, operating at 13.56 MHz, is essentially a short-range, two-way evolution of RFID. It allows a smartphone to both energize and read a wearable patch—for example, an NFC-based cortisol sensor scanned directly on skin (Sun et al., 2022a). These passive methods combine high security, simplicity, and comfort but are limited to a few centimeters.

BLE and Zigbee: BLE dominates consumer-grade wearables such as smart rings and watches. Operating at 2.4 GHz, it streams data continuously over 10–30 m with minimal power use, ideal for real-time heart rate or motion monitoring (Kong et al., 2024). Zigbee, by contrast, forms low-power mesh networks, linking multiple sensors into coordinated “body-area networks.” Though slower in data rate, its multi-node scalability suits rehabilitation suits and motion-tracking systems.

Rather than competing, these protocols now complement each other. RFID/NFC excel at short-range, battery-free sensing; BLE extends continuous real-time streaming; Zigbee enables distributed, multi-sensor intelligence. Hybrid systems—such as an NFC-powered patch that uploads data via BLE—illustrate the shift toward context-adaptive, connected biosensing (Kong et al., 2024). Future designs will merge these technologies to balance energy efficiency, user comfort, and data richness.

### 9.7 Integration of artificial intelligence (AI) and IoT: real-world applications

The convergence of Artificial Intelligence (AI) and the Internet of Things (IoT) with wearable biosensors for body-fluid analysis (sweat, interstitial fluid, tears, saliva) is reshaping precision diagnostics from passive monitoring to adaptive, predictive intelligence. Modern AI algorithms—particularly machine and deep learning—enhance signal reliability by filtering motion artifacts, baseline drift, and environmental noise inherent in body-fluid sensing. They enable multimodal data fusion across electrochemical, optical, and mechanical domains, extracting hidden biomarkers and physiological trends that traditional threshold-based methods miss (Kargarandehkordi et al., 2025; Zhang et al., 2023). For instance, AI-driven wearable networks have been shown to integrate sweat electrolyte data and motion cues to detect fatigue or dehydration with higher accuracy than linear statistical models (Huang et al., 2025).

IoT connectivity complements this intelligence by establishing continuous feedback loops between sensors, cloud servers, and clinicians, transforming single-use wearables into components of distributed health networks. Modular architectures combining local edge analytics with cloud computing minimize latency and energy consumption while maintaining scalability (Hosain et al., 2024). Real-world trials have demonstrated that IoT-enabled biosensors can remotely track hydration state, glucose variability, and renal stress in near real time, with automated alerts improving clinical responsiveness (Luo et al., 2024).

Despite this progress, the union of AI and IoT in chemical biosensing faces persistent challenges: limited generalizability of data-driven models across users and environments, energy constraints of on-device inference, and vulnerability of networked systems to synchronization failures and security breaches. Moreover, “black-box” algorithms undermine interpretability—an essential factor for clinical trust. Addressing these issues requires hybrid strategies coupling physics-based priors with data-driven learning, continual recalibration to correct for sensor drift, and on-device edge intelligence that ensures privacy and real-time decision-making. When properly integrated, AI supplies cognition and IoT provides communication—together enabling context-aware, connected biosensing. Yet, the impact of this synergy ultimately depends on sensor fidelity, calibration integrity, and algorithmic transparency. As research shifts toward scalable printed electronics, self-calibrating models, and secure, low-power networking, body-fluid biosensors are poised to evolve from passive monitors into intelligent companions for personalized healthcare.

## 10 Conclusion and future directions

Wearable sensors for body fluid monitoring have significantly transformed healthcare, sports science, and occupational health by providing real-time, non-invasive access to critical physiological data. These devices bridge the gap between traditional laboratory diagnostics and continuous, on-the-go health monitoring. Body fluids such as blood, sweat, saliva, and urine serve as rich sources of biomarkers, offering insights into metabolic health, hydration, and overall wellbeing.

The integration of advanced materials like PDMS, TPU, and graphene, along with manufacturing techniques such as 3D printing and stereolithography, has been instrumental in advancing wearable sensor technology. These innovations have enabled the development of flexible, durable, and sensitive devices that can adapt to various applications. Wearable sensors have demonstrated significant utility in domains ranging from sports science—where they optimize performance and recovery—to healthcare, where they monitor chronic conditions and support early disease detection. Furthermore, they play a crucial role in ensuring safety and efficiency in high-risk professions by tracking hydration and stress levels.

Despite these advancements, wearable sensor technology faces challenges that limit widespread adoption. Ensuring accuracy and reliability across diverse environments and skin types remains a technical hurdle. Power supply limitations and concerns over data security in wireless communication systems like RFID and Bluetooth also need to be addressed. Additionally, while advanced manufacturing methods have improved affordability, scaling production to meet global demand efficiently requires further innovation.

An increasing emphasis on environmental sustainability is reshaping the development of wearable sensors. The adoption of biodegradable and recyclable materials, such as cellulose-based substrates and graphene, addresses environmental concerns while ensuring long-term viability. Energy harvesting technologies, like triboelectric nanogenerators (TENGs), promise self-powered sensors that minimize reliance on disposable batteries, reducing electronic waste. These advancements align with global efforts to promote a circular economy and environmentally conscious technology (Zhang et al., 2025).

Artificial intelligence (AI) has emerged as a transformative force in wearable sensor technology, enabling smarter and more efficient health monitoring. AI algorithms can process vast amounts of sensor data in real time, providing actionable insights and predictive analytics. For instance, machine learning models can analyze patterns in body fluid biomarkers to predict hydration status, detect early signs of metabolic disorders, or assess recovery rates in athletes. AI-powered wearable systems can personalize health recommendations by integrating contextual information such as user activity levels, environmental conditions, and historical health data. Moreover, advanced AI techniques like deep learning enhance sensor calibration and fault detection, improving reliability and reducing errors caused by environmental or usage variability.

Moving forward, interdisciplinary collaboration among materials scientists, engineers, and healthcare professionals will play a pivotal role in driving sustainability. Sustainable innovations not only enhance device functionality but also contribute to user experience by increasing durability and facilitating end-of-life recycling. However, challenges remain in balancing production costs, supply chain logistics, and consumer awareness to achieve widespread adoption of eco-friendly materials and energy-efficient designs. Moreover, sustainable manufacturing practices can help scale production efficiently to meet growing global demand, ensuring wearable sensors contribute positively to both health outcomes and environmental preservation.

In the long term, wearable sensors are expected to evolve into fully integrated systems capable of monitoring multiple biomarkers, communicating with healthcare providers, and delivering therapeutic interventions. These systems will play a pivotal role in the transition toward personalized and preventive medicine, empowering individuals to take proactive control of their health. By addressing existing challenges, embracing sustainable materials, and integrating cutting-edge innovations, wearable sensors have the potential to revolutionize health monitoring, creating a healthier, more connected, and environmentally responsible future.
